# Bone Health in the Transgender and Gender Diverse Youth Population

**DOI:** 10.1007/s11914-023-00799-2

**Published:** 2023-07-03

**Authors:** Janet Y. Lee

**Affiliations:** 1 Department of Pediatrics, Division of Pediatric Endocrinology, University of California, San Francisco, San Francisco, CA 94143 USA; 2 Department of Medicine, Division of Endocrinology & Metabolism, University of California, San Francisco, San Francisco, CA 94143 USA; 3 Endocrine and Metabolism Section, San Francisco Veterans Affairs Health Care System, San Francisco, CA USA

**Keywords:** Transgender and gender diverse youth, Bone health, Gender-affirming medical therapy, Gonadotropin-releasing hormone agonist, Gender-affirming hormone therapy

## Abstract

**Purpose of Review:**

The purpose of this review is to summarize the scientific evidence on bone health in transgender and gender diverse (TGD) youth.

**Recent Findings:**

Gender-affirming medical therapies may be introduced during a key window of skeletal development in TGD adolescents. Before treatment, low bone density for age is more prevalent than expected in TGD youth. Bone mineral density *Z*-scores decrease with gonadotropin-releasing hormone agonists and differentially respond to subsequent estradiol or testosterone. Risk factors for low bone density in this population include low body mass index, low physical activity, male sex designated at birth, and vitamin D deficiency. Peak bone mass attainment and implications for future fracture risk are not yet known.

**Summary:**

TGD youth have higher than expected rates of low bone density prior to initiation of gender-affirming medical therapy. More studies are needed to understand the skeletal trajectories of TGD youth receiving medical interventions during puberty.

## Introduction

The transgender and gender diverse (TGD) youth population presenting to clinics worldwide has grown [1–5], with survey-based estimates of 1.6% youth population aged 13 to 17 years in the USA and 0.73% youth population aged 15 to 19 years in Canada identifying as transgender or gender non-binary [6, 7]. Clinical practice guidelines have included recommendations on care of TGD youth, with consideration of gender-affirming medical therapy in youth with significant gender dysphoria [8] by utilizing the potential intervention of gonadotropin-releasing hormone agonist (GnRHa) therapy as early as the onset of puberty to allow for exploration of gender without development of unwanted secondary sexual characteristics, followed by discontinuation of GnRHa or addition of gender-affirming hormone therapy (GAHT) with estradiol or testosterone by 16 years of age [9, 10]. Progestins such as lynestrenol and anti-androgens such as cyproterone acetate, bicalutamide, and spironolactone have also been used in the TGD youth population for menstrual and androgen suppression, respectively [9–14].

Since gender-affirming medical therapies may be prescribed during the critical window of peak bone mass accrual, attention has turned toward bone health in TGD youth. Because pediatric gender-affirming hormone therapies were first introduced in 1997 in the Netherlands [15], and in 2007 in the USA [16], long-term outcomes such as peak bone mass attainment and fracture risk have yet to be published. There are two notable approaches to timing the initiation of GAHT, with the “Dutch protocol” starting at 15 to 16 years of age [17], and a “peer-concordant puberty timing model” starting within the usual puberty timing window by 14 years of age, and oftentimes earlier [18].

## Measurement and Interpretation of Bone Mass in TGD Youth

To date, there is no official guidance for interpretation of areal bone mineral density (aBMD) by dual-energy X-ray absorptiometry (DXA) in TGD youth. For pediatric patients, lumbar spine (LS) and total body less head (TBLH) sites are preferred for DXA over the total hip (TH) and femur neck (FN) sites [19]. The International Society of Clinical Densitometry (ISCD) has provided guidance on interpretation of DXA in TGD adults, recommending aBMD *Z*-scores concordant with gender identity in transgender adults and aBMD *Z*-scores concordant with sex designated at birth in non-binary adults [19, 20]. Most literature on bone measures in TGD individuals report aBMD and bone mineral apparent density (BMAD) *Z*-scores concordant with sex designated at birth, with noteworthy exceptions: a long-term follow-up report including aBMD *Z*-scores for both sex references in the first known TGD individual to receive GnRHa and GAHT in adolescence [21]; a prospective study of proandrogenic and antiandrogenic progestins in late pubertal TGD youth reported aBMD *Z*-scores using both sex references [13]; a retrospective study of late pubertal transgender boys designated female at birth (DFAB) examined BMAD *Z*-scores using both sex references [22]; a retrospective study of mostly late pubertal TGD youth reporting aBMD *Z*-scores using both sex references [23]; a prospective study specifically exploring different methodologies of DXA interpretation in early pubertal TGD youth based on both sex references and chronologic or bone age [24•].

Given literature demonstrating that hip bone geometry metrics subperiosteal width and endocortical diameter matched affirmed gender curves in TGD individuals if GnRHa was initiated in early, rather than in mid or late, puberty [25•], TGD youth who initiate gender-affirming medical therapies earlier in puberty (Table 1) follow skeletal trajectories distinct from TGD youth who initiate gender-affirming medical therapies later in puberty (Table 2).

**Table 1:** Studies of bone measures in early to mid-pubertal (Tanner stage 2–3) TGD youth

Author; year	Type of study	Intervention	Participants	Follow-up	Results
Vlot, 2017	Retrospective	GnRHa, GAHT	*N* = 56 (22 DMAB, 34 DFAB) 16 DMAB bone age < 15 years 11 DFAB bone age < 14 years	aBMD & BMAD by DXA before GnRHa, on GnRHa before GAHT, and after 24 months of GAHT; BTMs (P1NP, ICTP, OC)	FN BMAD *Z*-scores increased after 24 months of GAHT in younger DFAB LS BMAD *Z*-scores decreased on GnRHa and increased after 24 months of GAHT in younger TGD cohort Younger TGD cohort overall had higher concentrations of BTMs before GnRHa. P1NP and ICTP decreased after GnRHa. P1NP decreased in younger DMAB and ICTP decreased in younger DFAB after 24 months of GAHT. No changes in OC were observed
Schagen, 2020	Prospective observational	None, GnRHa, GAHT	*N* = 121; *N* = 29 Tanner stage 2–3 (15 DMAB, 14 DFAB)	aBMD and BMAD by DXA before GnRHa, on GnRHa before GAHT (2.5 years DMAB and 4 years DFAB), and after GAHT (36 months)	DMAB had lower baseline BMD and BMAD *Z*-scores than DFAB participants BMD and BMAD *Z*-scores decreased over 24 months of GnRHa, with smaller incremental decreases between 24 and 36 months of GnRHa Mean BMD and BMAD *Z*-scores increased to above baseline after GAHT in all TGD youth
Lee, 2020	Prospective observational	None	*N* = 63 (33 DMAB, 30 DFAB) 63.5% Tanner stage 2 36.5% Tanner stage 3	aBMD by DXA and vBMD by QCT at Baseline only	Low BMD in 30% DMAB and 13% DFAB participants Low BMD participants had lower physical activity than normal BMD participants ( +)predictors of TH aBMD *Z*-scores: DFAB, 25-OHD ( +)predictor of TBLH aBMD *Z*-scores: BMI *Z*-score (-)predictors of TH & FN aBMD *Z*-scores: later age at GnRHa
Guss, 2022	Prospective observational	None	*N* = 6 (all DMAB) All Tanner stage 2–3	aBMD by DXA, and BMAT by MRI at Baseline only	Low BMD in 50% at TBLH and 33% at LS T1-R and MRS fat fraction not corelated with DXA measures
Lee, 2022	Prospective observational	None	*N* = 35 (18 DMAB, 17 DFAB) 65.7% Tanner stage 2 34.3% Tanner stage 3	aBMD by DXA	Low BMD in 44.4% DMAB and 11.8% DFAB
Marwa, 2022	Retrospective	None (*N* = 31), GnRHa, and/or GAHT (*N* = 88)	*N* = 119 (46 DMAB, 73 DFAB) (12 DMAB and 6 DFAB Tanner stage 2–3)	aBMD by DXA before or within 180 days of starting GnRHa and/or GAHT	No apparent differences between DMAB and DFAB LS aBMD *Z*-scores in early puberty participants
Nasomyont, 2022	Prospective observational	GnRHa	*N* = 6 (4 DMAB, 2 DFAB) and 3 cisgender females 100% Tanner stage 2	aBMD & BMAD by DXA, vBMD by pQCT, and BMAT by MRI at Baseline and 12 months	LS aBMD HAZ, TBLH aBMD HAZ, LS BMAD *Z*-score and trabecular vBMD decreased in TGD participants & increased in cisgender participants. Cortical vBMD increased in TGD participants T1-R declined and MRS fat fraction increased in TGD participants

*aBMD*, areal bone mineral density; *BMAD*, bone mineral apparent density; *BMAT*, bone marrow adipose tissue; *BMD*, bone mineral density; *BMI*, body mass index; *BTMs*, bone turnover marker; *DFAB*, designated female at birth; *DMAB*, designated male at birth; *DXA*, dual-energy X-ray absorptiometry; *FN*, femur neck; *GnRHa*, gonadotropin-releasing hormone agonist; *HAZ*, height for age adjusted aBMD *Z*-scores; *ICTP*, cross-linked telopeptide of type I collagen; *LS*, lumbar spine; *MRI*, magnetic resonance imaging; *MRS*, magnetic resonance spectroscopy; *OC*, osteocalcin; *P1NP*, procollagen type 1 N-terminal propeptide; *pQCT*, peripheral quantitative computed tomography; *QCT*, quantitative computed tomography; *TGD*, transgender and gender diverse; *T1-R*, T1 relaxation; *TBLH*, total body less head; *TH*, total hip; *vBMD*, volumetric bone mineral density

**Table 2:** Studies of bone measures in mid- to late pubertal (Tanner stage 4–5) TGD youth

Author; year	Type of study	Intervention	Participants	Follow-up	Results
Klink, 2015	Retrospective	GnRHa, GAHT	*N* = 34 (15 DMAB, 19 DFAB) Tanner stage 4–5	aBMD & BMAD by DXA before GnRHa, on GnRHa before GAHT, and after GAHT	DMAB participants had lower baseline LS and FN aBMD & BMAD *Z*-scores than DFAB participants LS BMD *Z*-scores decreased from baseline to median 5.8 years of GAHT in DMAB LS aBMD & BMAD *Z*-scores and FN aBMD *Z*-scores decreased on GnRHa monotherapy in DFAB LS BMAD *Z*-scores and FN *Z*-scores increased from start of GAHT to median 5.4 years of GAHT in DFAB LS aBMD *Z*-scores increased from baseline to median 5.4 years of GAHT in DFAB
Vlot, 2017	Retrospective	GnRHa, GAHT	*N* = 56 (22 DMAB, 34 DFAB) 6 DMAB bone age $\ge$ 15 years 23 DFAB bone age $\ge$ 14 years *12 participants from Klink, 2015 cohort	aBMD & BMAD by DXA before GnRHa, on GnRHa before GAHT, and after 24 months of GAHT BTMs (P1NP, ICTP, OC)	FN and LS BMAD *Z*-scores decreased on GnRHa and increased after 24 months of GAHT in older DFAB LS BMAD *Z*-scores increased after 24 months of GAHT in older DMAB Older TGD cohort overall had lower concentrations of BTMs before GnRHa. P1NP decreased after GnRHa in older DMAB and decreased after 24 months of GAHT in the older TGD cohort. OC increased after GnRHa and decreased after 24 months of GAHT in older DFAB. No changes in ICTP were observed
Tack, 2018	Prospective	Cytoproterone acetate, lynestrenol	*N* = 65 (21 DMAB, 44 DFAB) Tanner stage 4–5	aBMD by DXA pre-progestin treatment and post-progestin treatment (mean 11.64 months lynestrenol and mean 10.57 months cytoproterone acetate) vBMD, bone geometry, and SSIp by pQCT at distal radius and tibia BTMs (P1NP, s-CTX)	All aBMD *Z*-scores were negative at baseline TH aBMD *Z*-scores increased in DFAB participants Whole body, LS, TH, and FN aBMD *Z*-scores decreased in DMAB participants All pQCT trabecular and cortical bone parameters at distal radius and tibia increased similar to age-matched cisgender female controls in DFAB participants Trabecular vBMD decreased at distal radius and tibia, total and trabecular vBMD *Z*-scores decreased at distal radius, and cortical BMC and vBMD increased at midshaft tibia and radius SSIp *Z*-scores decreased significantly at midshaft radius in DMAB participants Periosteal circumference *Z*-scores decreased in DMAB participants P1NP decreased by 9.3% in DFAB and 46.5% in DMAB. S-CTX decreased by 17% in DMAB
Stoffers, 2019	Retrospective	GnRHa, GAHT	*N* = 62 (all DFAB) 91% post-menarche	aBMD & BMAD by DXA pre-GnRHa, pre-GAHT (median 8 months GnRHa), then every 1–2 years (median 12 months GAHT)	LS and FN aBMD *Z*-scores decreased on GnRHa monotherapy LS and FN BMD *Z*-scores remained lower than baseline after 12 and 24 months of GAHT LS BMAD *Z*-scores were lower than baseline after GAHT
Joseph, 2019	Retrospective	GnRHa	*N* = 70 (31 DMAB 43% late pubertal, 39 DFAB 94.9% post-menarche)	aBMD & BMAD by DXA pre-GnRHa and after 1 and 2 years of GnRHa	Baseline LS and FN aBMD and LS BMAD *Z*-scores were lower in (older) DFAB than (younger) DMAB participants LS and FN aBMD and LS BMAD *Z*-scores decreased after 1 and 2 years of GnRHa
Schagen, 2020	Prospective observational	None, GnRHa, GAHT	*N* = 121; *N* = 92 Tanner stage 4–5 (36 DMAB, 56 DFAB)	aBMD and BMAD by DXA before GnRHa, on GnRHa before GAHT (1.5 years DMAB and 1.7 years DFAB), and after GAHT (36 months)	BMAD *Z*-scores were higher in DFAB participants than in DMAB participants LS, FN, and TBLH aBMD *Z*-scores and LS and FN BMAD *Z*-scores decreased after GnRHa monotherapy LS, FN, TBLH aBMD *Z*-scores increased after GAHT in late pubertal DFAB participants LS BMAD *Z*-scores increased after GAHT in all late pubertal TGD youth FN BMAD *Z*-scores increased after GAHT in late pubertal DMAB participants
Navabi, 2021	Retrospective	None, GnRHa	*N* = 172 (baseline—119 transgender males DFAB 90.7% Tanner stage 4–5, 51 transgender females DMAB 80.3% Tanner stage 4–5, 2 non-binary—not included in DXA data) Pre- and post-GnRHa (36 DMAB and 80 DFAB)	aBMD & BMAD by DXA pre- and post-GnRHa for 355.2 $\pm$ 96.7 days	DMAB had lower baseline LS aBMD & BMAD *Z*-scores, TH aBMD *Z*-scores, and BMC *Z*-scores than DFAB participants LS, TH, TBLH aBMD *Z*-scores decreased post-GnRHa in all TGD youth. LS BMAD *Z*-scores decreased post-GnRHa in DMAB participants 3 DMAB and 1 DFAB had low baseline aBMD at LS 55.2% of cohort had vitamin D deficiency or insufficiency Vitamin D status was associated with baseline LS aBMD & BMAD *Z*-scores and L TH aBMD *Z*-scores
Marwa, 2022	Retrospective	None (*N* = 31), GnRHa, and/or GAHT (*N* = 88)	*N* = 119 (46 DMAB, 73 DFAB) (34 DMAB and 67 DFAB Tanner stage 4–5)	aBMD by DXA before or within 180 days of starting GnRHa and/or GAHT	Lower LS aBMD *Z*-scores in DMAB than in DFAB later puberty participants ( +)predictor of LS aBMD *Z*-score: BMI *Z*-score (-)predictor of LS aBMD *Z*-score: vitamin D deficiency
Hodax, 2022	Retrospective	None	*N* = 64 (47 DMAB, 17 DFAB) Mean age 14.2 $\pm$ 2.5 years	aBMD by DXA	DMAB had lower aBMD *Z*-scores than DFAB participants Low aBMD at TBLH in 30% of cohort Low aBMD at TBLH in 36% DMAB and 12% DFAB participants Low aBMD at LS in 17% DMAB and 5% DFAB participants ( +)association of BMI *Z*-scores to aBMD *Z*-scores

*aBMD*, areal bone mineral density; *BMAD*, bone mineral apparent density; *BMAT*, bone marrow adipose tissue; *BMD*, bone mineral density; *BMI*, body mass index; *BTMs*, bone turnover marker; *DFAB*, designated female at birth; *DMAB*, designated male at birth; *DXA*, dual-energy X-ray absorptiometry; *FN*, femur neck; *GnRHa*, gonadotropin-releasing hormone agonist; *HAZ*, height for age adjusted aBMD *Z*-scores; *ICTP*, cross-linked telopeptide of type I collagen; *LS*, lumbar spine; *MRI*, magnetic resonance imaging; *MRS*, magnetic resonance spectroscopy; *OC*, osteocalcin; *P1NP*, procollagen type 1 N-terminal propeptide; *pQCT*, peripheral quantitative computed tomography; *QCT*, quantitative computed tomography; *s-CTX*, serum C-terminal telopeptide; *SSIp*, polar strength strain index; *TGD*, transgender and gender diverse; *T1-R*, T1 relaxation; *TBLH*, total body less head; *TH*, total hip; *vBMD*, volumetric bone mineral density

## BMD in TGD Youth

As more studies focused on the skeletal effects of gender-affirming medical therapy in TGD youth emerge, there is still much to be explored in this field. The initial studies came from the pioneering Dutch, and subsequent studies have now been published across the world. However, long-term studies focused on older TGD individuals who began gender-affirming medical therapy in their pubertal years have yet to be published given the relative recent provision of pediatric gender-affirming medical care. It remains to be seen whether the “Dutch protocol” and “peer-concordant puberty timing model” affect ultimate peak bone mass attainment differently.

### Early to Mid-Pubertal (Tanner Stage 2–3) TGD Youth

In early to mid-pubertal TGD youth who have not initiated gender-affirming medical therapy, multiple groups have reported lower aBMD and BMAD *Z*-scores in transfeminine youth who were designated male at birth (DMAB) than in transmasculine youth who were DFAB [24•, 26•, 27•]. One study included a majority of early pubertal TGD youth DMAB (*n* = 31, 57% Tanner stage 2–3) but did not separate data based on pubertal status [28].

The first study to report DXA data differentially based on pubertal status in TGD adolescents retrospectively utilized bone age cut-offs of < 15 years for DMAB and < 14 years for DFAB to define a younger cohort. This study included 42 DFAB and 28 DMAB individuals with varying numbers of participants who had DXA scans prior to GnRHa, prior to GAHT, and after 24 months of GAHT. The younger DMAB TGD youth had negative FN BMAD *Z*-scores at baseline through 24 months of GAHT; at 24 months of GAHT, younger DMAB TGD youth had lower FN BMAD *Z*-scores than younger DFAB TGD youth. At baseline, LS BMAD *Z*-scores were lower in younger DMAB TGD youth than younger DFAB TGD youth. The younger cohort had decreased FN and LS BMAD *Z*-scores from baseline to start of GAHT, a period of GnRHa monotherapy; in the younger TGD youth, FN and LS BMAD *Z*-scores increased after 24 months of GAHT but remained lower than baseline [26•].

One multi-site prospective study of 63 early pubertal (63.5% Tanner stage 2) TGD youth (52.4% DMAB) utilizing DXA and quantitative computed tomography (QCT) imaging modalities showed higher than expected prevalence of low bone density (at least one areal or volumetric BMD *Z*-score ≤  − 2) prior to initiation of any gender-affirming medical interventions: 30% in DMAB and 13% in DFAB participants [27•]. Additionally, the weight-bearing cortical bone-rich hip sites were the only sites with statistically significant differences in aBMD *Z*-scores between DMAB and DFAB participants. Prospective collection of dietary calcium intake, vitamin D status, and physical activity assessment demonstrated that TGD youth who had low bone density had statistically significantly lower scores on the Physical Activity Questionnaire for Older Children than TGD youth with normal bone density, 2.32 ± 0.71 vs. 2.76 ± 0.61 (*p* = 0.01), and dietary calcium intake was suboptimal in the entire cohort [27•]. Multivariate linear regression revealed body mass index (BMI) *Z*-score to be a positive predictor of TBLH aBMD *Z*-scores. Female sex designated at birth and serum 25-hydroxyvitamin D were positive predictors and age at GnRHa initiation was a negative predictor of TH and FN aBMD *Z*-scores [27•].

An observational prospective study which included 29 early to mid-pubertal TGD youth (15 DMAB and 14 DFAB) found lower pre-treatment aBMD and BMAD *Z*-scores in DMAB than in DFAB, as well as expected decreases in aBMD and BMAD *Z*-scores over 24 months of GnRHa monotherapy (2.5 years in DMAB and 4.0 years in DFAB), with smaller incremental decreases in aBMD and BMAD *Z*-scores in the following year for those who continued for 36 months of GnRHa monotherapy (11 DMAB and 4 DFAB) [29•]. In the 15 early to mid-pubertal TGD youth who went on to receive 36 months of GAHT (10 DMAB and 5 DFAB), mean aBMD and BMAD *Z*-scores reassuringly increased to higher than baseline [29•].

A small prospective case series of six early to mid-pubertal transgender girls DMAB prior to GnRHa reported that half had low bone density at TBLH, and one-third had low bone density at LS; notably, half of these youth had BMI < 18 kg/m^2^ [30]. Another prospective study of 35 early pubertal (65.7% Tanner stage 2) TGD youth prior to initiating GnRHa also reported substantial percentages of low bone density in 44.4% of DMAB participants and in 11.8% of DFAB participants [24•].

A subset of 18 TGD youth (12 DMAB and 6 DFAB) in Tanner stage 2–3 of puberty in a larger retrospective cohort of TGD youth (*n* = 119) who had DXA scans before or within 180 days of initiating GnRHa and/or GAHT showed no statistically significant differences in LS aBMD *Z*-scores between DMAB and DFAB participants [23].

These studies have demonstrated that early to mid-pubertal DMAB TGD youth have higher incidence of low bone density and lower aBMD and BMAD *Z*-scores than DFAB youth prior to initiation of GnRHa and/or GAHT. Only shorter term studies of bone measures have examined skeletal trajectories of these early to mid-pubertal TGD youth after 2 to 3 years of GAHT [26•, 29•].

### Mid- to Late Pubertal (Tanner Stage 4–5) TGD Youth

The first group to report retrospective bone measures in TGD adolescents showed lower aBMD and BMAD *Z*-scores in DMAB than in DFAB who were in later puberty (Tanner 4–5) prior to starting GnRHa therapy, with expected decreases in aBMD and BMAD *Z*-scores for all TGD adolescents on GnRHa monotherapy for median 1.3 and 1.5 years in DMAB and DFAB, respectively [31•]. After a median duration of 5.8 and 5.4 years of GAHT in DMAB and DFAB, respectively, aBMD and BMAD *Z*-scores were still lower than pre-treatment values, although the decreases in the aBMD and BMAD *Z*-scores of the DMAB were more notable and included scores in the low bone density range. The notable decreases in the DMAB were only statistically significant in the LS aBMD *Z*-scores from baseline to median 5.8 years GAHT [31•]. A later study that included 12 participants from this original cohort of TGD adolescents has shown similar trends of lower aBMD and BMAD *Z*-scores in DMAB that did not increase back to baseline after 24 months of GAHT except in the older DMAB participants [26•].

A prospective study of 65 late pubertal (Tanner stage 4–5) TGD youth (21 DMAB and 44 DFAB) who received antiandrogenic and proandrogenic progestins cytoproterone acetate and lynestrenol, respectively, utilized DXA to assess aBMD *Z*-scores before and after progestin treatment. DMAB youth had mean 10.57 (range 5–31) months on cytoproterone acetate and DFAB youth had mean 11.64 (range 4–40) months on lynestrenol between DXA scans. All TGD youth had negative whole body, LS, TH, and FN aBMD *Z*-scores prior to progestin treatment. For DFAB participants, TH aBMD *Z*-scores increased significantly, and the remainder of the aBMD *Z*-scores did not change significantly. When DFAB participants were compared with age-matched male references, aBMD *Z*-scores decreased significantly at FN, LS, and whole body. For DMAB participants, aBMD *Z*-scores decreased significantly at all sites. When DMAB participants were compared with age-matched female references, aBMD *Z*-scores decreased at FN but not significantly at LS or whole body [13].

In a retrospective study focused on 62 DFAB TGD youth (91% post-menarche) receiving GnRHa (median eight months, range 3–39 months) and testosterone-based GAHT (median 12 months, range 5–33 months), DXA scans demonstrated lower aBMD *Z*-scores at LS and FN after GnRHa monotherapy. After 12 (*n* = 37) and 24 months (*n* = 15) of testosterone therapy, aBMD *Z*-scores remained lower than baseline values. LS and FN BMAD at 12 and 24 months of testosterone therapy were not significantly different from baseline BMAD. The LS BMAD *Z*-scores were significantly lower than baseline BMAD Z-scores, and usage of male reference *Z*-scores increased the BMAD *Z*-scores but still showed similar changes after testosterone therapy [22].

Another retrospective review of 70 TGD youth who were majority late pubertal (31 DMAB 43% late pubertal, 39 DFAB 94.9% post-menarche) examined changes in aBMD and BMAD by DXA before and after GnRHa. Unlike most of the other studies, baseline aBMD and BMAD *Z*-scores were lower in DFAB than DMAB participants; however, the DFAB cohort was in later puberty than the DMAB cohort, which was majority early pubertal. The LS and FN aBMD *Z*-scores and LS BMAD *Z*-scores decreased after 1 year of GnRHa in all TGD youth. In 10 DMAB and 21 DFAB who had DXA scans before and after 2 years of GnRHa, LS and FN aBMD *Z*-scores and LS BMAD Z-scores decreased from baseline in all TGD youth, although seemed to plateau after the first year of GnRHa [28].

A prospective observational cohort which included 92 late pubertal (Tanner stage 4–5) TGD youth (36 DMAB and 56 DFAB) who had DXA scans prior to GnRHa, on GnRHa (mean 1.5 years DMAB and 1.7 years DFAB) prior to GAHT, and after GAHT. BMAD *Z*-scores were generally higher in DFAB than in DMAB participants. LS, FN, and TBLH aBMD *Z*-scores and LS and FN BMAD *Z*-scores decreased significantly in all later pubertal TGD youth after 24 months of GnRHa monotherapy. LS, FN, and TBLH aBMD *Z*-scores increased significantly after 3 years of GAHT in late pubertal DFAB participants. LS BMAD *Z*-scores increased significantly after 3 years of GAHT in all late pubertal TGD youth, and FN BMAD *Z*-scores increased significantly after 3 years of GAHT in the late pubertal DMAB participants [29•].

A retrospective cohort of 172 mostly later pubertal TGD youth (119 DFAB 90.7% Tanner stage 4–5, 51 DMAB 80.3% Tanner stage 4–5, two non-binary) had serum vitamin D and DXA scans before and after GnRHa. Prior to GnRHa, the majority (55.2%) of the cohort had vitamin D deficiency or insufficiency, and vitamin D status was associated with baseline LS and TH aBMD *Z*-scores, and LS BMAD *Z*-scores. At baseline, DMAB participants had lower aBMD and BMAD *Z*-scores and bone mineral content (BMC) than DFAB participants. A subgroup of 36 DMAB and 80 DFAB had DXA scans before and after GnRHa, with a mean interval between pre- and post-DXA scans of 406.7 $\pm$ 98.3 days (range 210–720 days). LS, TH, and TBLH aBMD *Z*-scores decreased significantly in all TGD youth, and LS BMAD *Z*-scores decreased significantly in DFAB participants. At baseline, three DMAB and one DFAB had low bone density, and 20 DFAB had more than 1 standard deviation decrease in LS aBMD *Z*-score post-GnRHa [32].

A retrospective study of LS aBMD *Z*-scores collected from non-standardized DXA machines in a cohort of 119 TGD adolescents (46 DMAB and 73 DFAB) who were mostly late pubertal (Tanner 4–5 in 73.9% DMAB and 91.3% DFAB) showed statistically significant (*p* = 0.010) lower LS aBMD Z-scores in DMAB (− 0.605 ± 1.42) compared with DFAB (0.043 ± 1.09) prior to or within 180 days of starting GnRHa and/or GAHT, although post hoc analysis of the 31 TGD adolescents who had DXA scans before gender-affirming medical therapies did not have statistically significant differences (*p* = 0.077) in LS aBMD *Z*-scores between DMAB (− 0.58 ± 1.36) and DFAB (0.25 ± 1.19) [23]. Multivariate regression models identified vitamin D deficiency and lower BMI *Z*-scores as significant determinants of lower LS aBMD *Z*-scores [23].

An additional retrospective study of 64 TGD youth that did not collect pubertal staging information but likely included mostly mid- to late pubertal individuals (DMAB mean age 15.0 ± 2.0 years, DFAB mean age 12.0 ± 2.4 years) demonstrated lower mean aBMD *Z*-scores at TBLH, LS, TH, and FN sites in DMAB when compared with DFAB, with the lower limit range TBLH aBMD *Z*-score of − 4.1 in DMAB [33]. Notably, both DMAB and DFAB groups included TGD youth who have low bone density, and the group also found a positive correlation between BMI *Z*-scores and aBMD *Z*-scores at all sites [33].

In mid- to late pubertal TGD youth, DMAB participants had lower aBMD and BMAD *Z*-scores than DFAB participants, similar to patterns observed in early to mid-pubertal TGD youth. These TGD youth have expected decreases in aBMD and BMAD *Z*-scores while on GnRHa or antiandrogenic progestin therapy, and studies have reported mixed results with respect to aBMD and BMAD *Z*-scores after short to medium term duration of GAHT.

## Quantitative Computed Tomography in TGD Youth

Various modalities of QCT have been utilized in a handful of studies focused on skeletal imaging in TGD youth. In 65 late pubertal TGD youth (21 DMAB and 44 DFAB) receiving cytoproterone acetate and lynestrenol, respectively, volumetric (vBMD) was assessed by peripheral (pQCT) at trabecular and cortical non-dominant radius (4% and 66% from distal) and left tibia (4% and 38% from distal) sites. At the 66% non-dominant radius site, polar strength strain index (SSIp) was calculated. All pQCT trabecular and cortical bone parameters at radius and tibia increased similarly to age-matched cisgender female controls in DFAB participants receiving lynestrenol. There were no changes in SSIp for DFAB participants receiving lynestrenol. In DMAB participants receiving cytoproterone acetate, trabecular vBMD decreased at distal radius and tibia, total, and trabecular vBMD Z-scores decreased at distal radius, and cortical BMC and vBMD increased at midshaft tibia and radius. SSIp *Z*-scores decreased significantly at the midshaft radius in DMAB participants. DMAB also had significantly lower periosteal circumference *Z*-scores over the study period [13].

An aforementioned study of 63 TGD early pubertal (Tanner stage 2–3) youth reported vBMD by QCT of lumbar spine and hip for trabecular and cortical vBMD, respectively, in 15 participants (eight DMAB and seven DFAB). Similar to the DXA findings of the 48 other participants who had BMD assessed by DXA, the DMAB participants had statistically significantly lower mean vBMD *Z*-scores at the primarily cortical bone hip than the DFAB participants, − 1.80 ± 1.42 vs. − 0.42 ± 0.92 (*p* = 0.047) [27•].

In the previously mentioned prospective study of six early pubertal (Tanner stage 2) TGD youth (four DMAB and two DFAB) compared with three cisgender female youth over a 12-month period of GnRHa therapy for the TGD youth and no treatment for the cisgender youth, pQCT of the left tibia at 3% and 66% of tibial length was analyzed for trabecular and cortical vBMD, respectively. In the 12-month period, trabecular vBMD (3% tibia) decreased in the TGD cohort on GnRHa and increased in the cisgender cohort, while cortical vBMD (66% tibia) increased in the TGD cohort on GnRHa and decreased in the cisgender cohort [34].

Preliminary high-resolution pQCT (HR-pQCT) data have been presented from 34 of the 35 early pubertal TGD youth whose baseline DXA measures have been published [24•, 35]. HR-pQCT bone measures of non-dominant distal radius and distal tibia metaphyses centered 4.0% proximal to the growth plate as well as strength estimates by micro-finite element analyses ($\mu$ FEA) were analyzed prior to initiation of GnRHa. Overall, low aBMD was associated with low bone strength as estimated by failure load using $\mu$ FEA and did not appear to be driven by bone size deficits. Low aBMD at the distal tibia predicted lower cortical and higher trabecular area, which suggested less compaction of bone at the weight-bearing site. Grip strength was a positive predictor at both distal radius and tibia sites, but recent physical activity was not a significant predictor. In addition, later age at social transition was a negative predictor of distal tibia failure load. These constellation of findings led authors to hypothesize that those who transitioned later had lower failure load possibly due to lower accumulated activity, as suggested by grip strength [35].

These early and forthcoming studies including QCT imaging may be able to shed more light on changes in bone microarchitecture and strength estimates in TGD youth treated with gender-affirming medical therapies during puberty.

## Bone Marrow Composition in TGD Youth

One small prospective case series evaluating bone marrow composition of six early to mid-pubertal (Tanner stage 2–3) transgender girls DMAB prior to GnRHa found that bone marrow magnetic resonance (MR) variables were not statistically significantly correlated with DXA measures, although the authors noted a non-significant correlation between higher R2 of water in bone marrow and increased eating disordered behavior as measured by the Eating Attitudes Test-26 (EAT-26) [30]. The same research group prospectively enrolled six early pubertal TGD youth (2 DFAB and 4 DMAB) and three early pubertal cisgender female participants to evaluate the effects of GnRHa compared with typical progression of puberty on bone marrow adipose tissues (BMAT) over a 12-month period, and found larger increases in BMAT indices in the TGD cohort compared with the cisgender cohort [34]. Larger studies are needed to evaluate BMAT changes in pubertal TGD youth prior to and after starting gender-affirming medical therapies.

## Bone Turnover Markers in TGD Youth

The first study reporting bone turnover marker (BTMs) data in TGD youth receiving GnRHa and GAHT included formation markers procollagen type 1 N-terminal propeptide (P1NP) and osteocalcin and resorption marker cross-linked telopeptide of type I collagen (ICTP) in younger and older cohorts based on bone age radiograph (DMAB < 15 years or $\ge$ 15 years and DFAB < 14 years or $\ge$ 14 years) at baseline prior to GnRHa, on GnRHa prior to GAHT, and after 24 months of GAHT. These BTMs, which were not specified to be drawn fasting, were mostly collected prior to start of GnRHa and GAHT, although some had BTMs drawn up to 32 days after GnRHa or 5 days after GAHT. In general, the younger TGD cohort had higher BTMs than the older TGD cohort. Osteocalcin did not seem affected by GnRHa or GAHT, although there was some increase in the older DFAB TGD cohort after GnRHa and subsequent decrease after 24 months of GAHT. P1NP and ICTP decreased after GnRHa in the younger TGD cohort [26•].

BTM P1NP and serum C-terminal telopeptide (s-CTX) were measured in a study of 65 late pubertal TGD youth (21 DMAB and 44 DFAB) receiving mean 10.57 (range 5–31) months of cytoproterone acetate and mean 11.64 (range 4–40) months of lynestrenol and demonstrated decreases from baseline values in P1NP of 9.3% in DFAB and 46.5% in DMAB participants. In DMAB participants, s-CTX also decreased by 17.1% when compared with pre-treatment values [13].

An additional study that included BTM data in TGD youth separated out cohorts based on pubertal stage and reported P1NP, osteocalcin, ICTP, and formation marker amino terminal of type III procollagen peptide (P3NP). Prior to GnRHa, there were no significant differences in serum levels of any of the BTMs between early and late pubertal DMAB. However, in the DFAB, all baseline BTMs were significantly higher in the early pubertal cohort than in the late pubertal cohort. All BTMs decreased after 2 years of GnRHa monotherapy in all DMAB and early pubertal DFAB. P3NP and ICTP significantly decreased in late pubertal DFAB. Prior to 3 years of GAHT, P1NP, P3NP, and ICTP were significantly higher in early pubertal DMAB than in late pubertal DMAB. In DFAB, baseline P1NP and P3NP were higher in the early puberty group than in the late puberty group. In the early pubertal DFAB and late pubertal DMAB, osteocalcin, P1NP, and P3NP decreased significantly in the first year of GAHT. Early pubertal DMAB had an initial increase in P1NP, P3NP, and ICTP in the first year of GAHT before decreasing after 2 and 3 years of GAHT. After 3 years of GAHT, all BTMs decreased significantly in DMAB participants. Osteocalcin, P1NP, and ICTP significantly decreased in DFAB participants [29•].

More studies are needed to understand the role of these BTM changes in TGD youth receiving gender-affirming medical therapies during puberty, and whether the subsequent increases after GAHT in TGD youth who receive GnRHa in earlier puberty are linked to changes in growth velocity.

## Other Risk Factors for Impaired Skeletal Health to Consider in TGD Youth

Other risk factors for impaired skeletal health such as minority stress [36, 37], decreased physical activity [38, 39], and disordered eating [40] are important considerations in TGD youth. Future studies focused on bone measures in TGD youth should include such factors in their analyses.

## Conclusions

The skeletal effects of gender-affirming medical therapy in TGD youth are complex and dependent on a variety of factors, including pubertal stage at time of initiation, timing, and duration of GnRHa and GAHT, as well other aspects. Data have shown that pre-treatment BMD *Z*-scores are lower in more TGD youth than expected based on the general population, affecting the DMAB population more than the DFAB population. Since BMD *Z*-scores typically drop with GnRHa, and in some studies continue decreasing with GAHT, identification of potential contributors to low baseline bone density, such as decreased physical activity, dietary calcium intake, or vitamin D status, are critical to mitigating the expected decrease in BMD *Z*-scores. Exciting developments including studies utilizing QCT, MR imaging, and BTMs may shed more light on implications of bone changes as we await further data on fracture risk. To date, most studies have included majority white and non-Hispanic participants, and forthcoming studies should strive to include more diverse cohorts of TGD youth. Longer-term studies are needed to determine ultimate peak bone mass attainment and how these bone measures influence current and future fracture risk.
